# Intertwined regulators: hypoxia pathway proteins, microRNAs, and phosphodiesterases in the control of steroidogenesis

**DOI:** 10.1007/s00424-024-02921-4

**Published:** 2024-02-15

**Authors:** Stephen Ariyeloye, Susanne Kämmerer, Erik Klapproth, Ben Wielockx, Ali El-Armouche

**Affiliations:** 1 Institute of Clinical Chemistry and Laboratory Medicine, Medical Faculty, Technische Universität Dresden, Fetscherstrasse 74, 01307 Dresden, Germany; 2 Department of Pharmacology and Toxicology, Medical Faculty, Technische Universität Dresden, Fetscherstrasse 74, 01307 Dresden, Germany

**Keywords:** Hypoxia, MicroRNA, Phosphodiesterases, Steroidogenesis, Regulation

## Abstract

Oxygen sensing is of paramount importance for maintaining cellular and systemic homeostasis. In response to diminished oxygen levels, the hypoxia-inducible factors (HIFs) orchestrate various biological processes. These pivotal transcription factors have been identified as key regulators of several biological events. Notably, extensive research from our group and others has demonstrated that HIF1α exerts an inverse regulatory effect on steroidogenesis, leading to the suppression of crucial steroidogenic enzyme expression and a subsequent decrease in steroid levels. These steroid hormones occupy pivotal roles in governing a myriad of physiological processes. Substantial or prolonged fluctuations in steroid levels carry detrimental consequences across multiple organ systems and underlie various pathological conditions, including metabolic and immune disorders. MicroRNAs serve as potent mediators of multifaceted gene regulatory mechanisms, acting as influential epigenetic regulators that modulate a broad spectrum of gene expressions. Concomitantly, phosphodiesterases (PDEs) play a crucial role in governing signal transduction. PDEs meticulously manage intracellular levels of both cAMP and cGMP, along with their respective signaling pathways and downstream targets. Intriguingly, an intricate interplay seems to exist between hypoxia signaling, microRNAs, and PDEs in the regulation of steroidogenesis. This review highlights recent advances in our understanding of the role of microRNAs during hypoxia-driven processes, including steroidogenesis, as well as the possibilities that exist in the application of HIF prolyl hydroxylase (PHD) inhibitors for the modulation of steroidogenesis.

## Hypoxia pathway proteins

Oxygen plays a central role in the complex world of cellular survival, serving as an essential molecule for all cells and tissues in our body. Its importance extends far beyond respiration, as oxygen is not only a critical component in the generation of cellular energy, but also serves as a key cofactor and substrate for a wide range of enzymes. The lack of sufficient oxygen, a condition known as hypoxia, can have profound effects on cellular function. However, it is important to realize that the concept of hypoxia is far from absolute, as what constitutes normal oxygen levels varies widely between different tissues. At a molecular level, the cellular adaptation to hypoxia is governed by a remarkable set of transcription factors known as hypoxia-inducible factors (HIFs). These hypoxia pathway proteins, including HIF1α and HIF2α, oversee the orchestration of hundreds of genes involved in adaptation processes, the preservation of cell viability, and interconnection to an enormous variety of biological functions [72, 89, 95, 120].

**Fig. 1:**
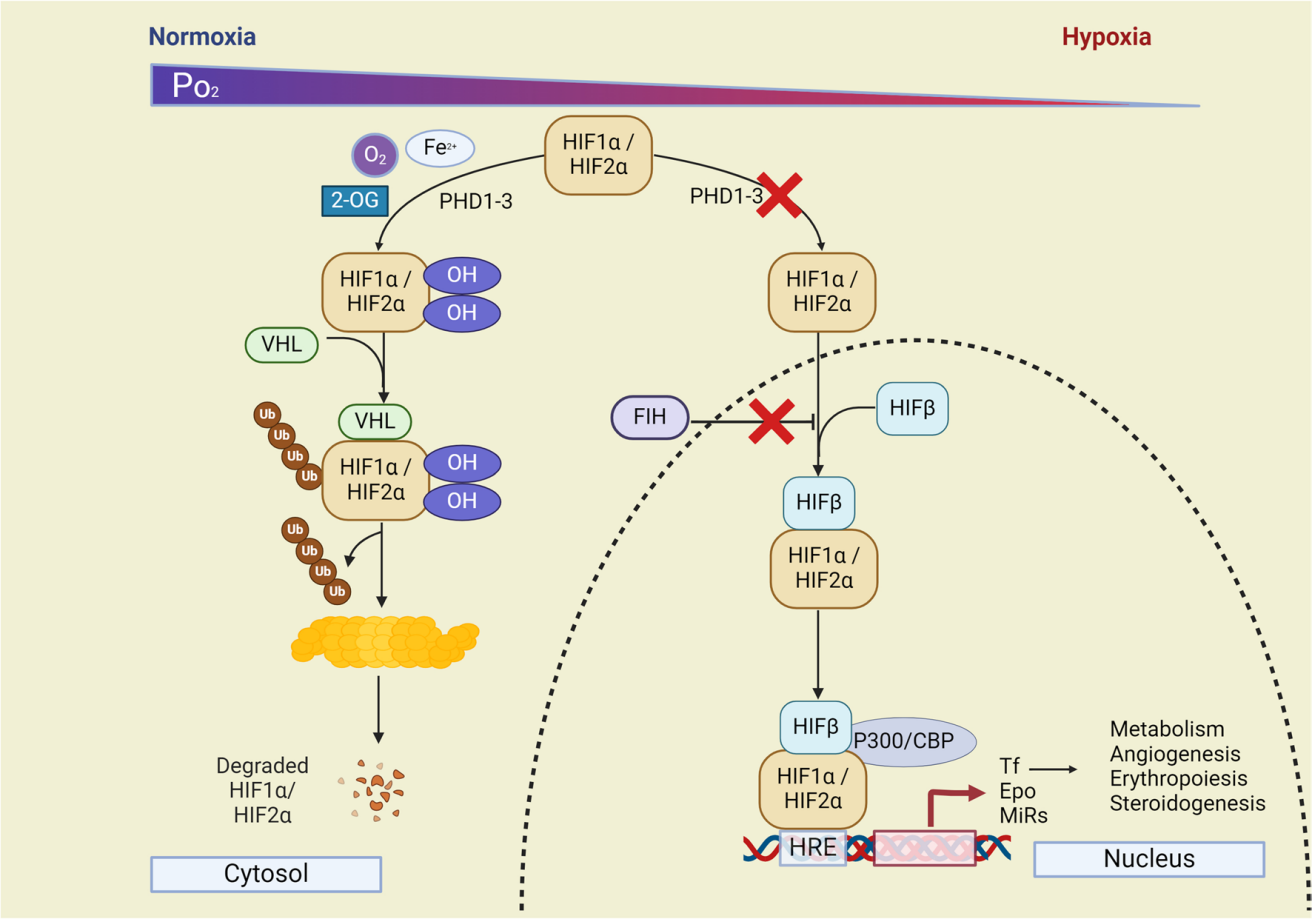
Hypoxia signaling pathway. Under normal oxygen levels (normoxia), hypoxia-inducible factor (HIF)-α subunits are hydroxylated by HIF prolyl hydroxylase domain proteins (PHDs). PHDs require oxygen (O_2_), ferrous iron (Fe^2+^), and 2-oxoglutarate (2-OG) for their activity. Hydroxylated HIFs are recognized and bound by the von Hippel-Lindau tumor suppressor protein (VHL). VHL recruits components of the E3-ubiquitin ligase complex and facilitates the ubiquitination and proteasomal degradation of hydroxylated HIFs. HIF is also regulated in a VHL-independent manner by the activity of factor inhibiting HIF1 (FIH), an asparaginyl hydroxylase. The conserved asparaginyl (Asn) residue within the C-terminal activation domain in HIFα is hydroxylated under normoxia or mild hypoxia, thereby suppressing HIF transcriptional activity by preventing interaction with the transcriptional coactivators p300/CBP. Conversely, under conditions of relatively low oxygen levels (hypoxia), PHD activity and the degradation of HIFα subunits as well as FIH activity are inhibited. Therefore, HIFα subunits are stabilized and are able to dimerize with the HIFβ subunit. In the nucleus, active HIFα/β dimer in complex with other cofactors promote the transcription of target genes with hypoxia response elements (HREs) such as transferrin (Tf), erythropoietin (Epo), numerous microRNAs, and other genes involved in metabolism, angiogenesis, erythropoiesis, steroidogenesis, and other hypoxia regulated processes

The structural backbone of these functional HIF transcription factors is a heterodimer composed of two subunits: an oxygen-sensitive HIFα subunit and a constitutively expressed HIF1β subunit [7, 111]. Under conditions of ample oxygen, HIF prolyl hydroxylase domain proteins (PHD1-3), known as oxygen sensors, step into the scene, hydroxylating the HIFα subunit at two specific proline residues within its oxygen-dependent degradation domain [6, 32]. Subsequently, hydroxylated HIFs are recognized and bound by the von Hippel-Lindau tumor suppressor protein (VHL). VHL recruits components of the E3-ubiquitin ligase complex and facilitates the ubiquitination and proteasomal degradation of hydroxylated HIFs (see Fig. 1). These PHD enzymes belong to the 2-oxoglutarate-dependent oxygenase superfamily and rely on oxygen, iron, and ascorbate for their enzymatic activity. However, in the oxygen-starved realm of hypoxic cells, PHDs remain inactive, allowing HIF1α or HIF2α to accumulate within the cell. These accumulated HIFs then partner with the HIF1β subunit in the nucleus, forming the functional HIF heterodimer accompanied by its co-activators (e.g., p300 and CBP). This nuclear engagement culminates in the formation of a transcriptional complex, with the HIF transcription factor binding to hypoxia-responsive elements (HRE) within gene promoters, which exerts regulatory control over numerous cellular processes, including erythropoiesis, angiogenesis, metabolism, and cellular proliferation and differentiation (see Fig. 1) [96].

Another oxygen-dependent mechanism of HIF regulation involves factor inhibiting HIF1 (FIH1), an asparaginyl hydroxylase, which hydroxylates the HIFα subunit at the asparagine in the C-terminal activation domain under normoxia and mild hypoxia. Such hydroxylation prevents the activation of HIF as it inhibits interactions between HIF1α and its p300/CBP co-activators [19]. FIH1 also acts as a safety net because it is less sensitive to a reduction in oxygen pressure compared to PHDs and remains active even under mild hypoxia to block HIFα activation that has escaped PHD-mediated degradation [49].

## MicroRNAs

MicroRNAs (miRNAs) are a class of endogenous, small, noncoding, single-stranded ribonucleic acids (RNAs) that are usually about 21–24 nucleotides in length and function in gene regulation. They repress the expression of their target genes post-transcriptionally and act as potent epigenetic regulators of a vast array of gene expressions [5]. Although still at its infancy state, microRNAs are implicated in myriads of biological processes as they underlie the regulation of processes such as metabolism, apoptosis, embryonic development, proliferation and differentiation, inflammation, and homeostasis amongst other physiological processes [105]. Furthermore, the aberrant expressions of miRNAs play critical roles in the pathogenesis of several diseases such as cancer, cardiovascular diseases, hepatitis, and many metabolic diseases. Amongst other types of noncoding RNAs (ncRNAs), miRNAs have emerged as key regulators of gene expression [2, 5, 26].

The biogenesis of miRNAs has been extensively described [81, 84, 129]. MiRNAs functionally regulate gene expression by base pairing partially or completely with complementary sites in their target messenger RNAs (mRNAs). The binding of miRNAs to their target mRNAs, usually in the 3′-untranslated region (3′UTR), triggers various mechanisms of miRNA gene regulation, such as alteration of mRNA stability, mRNA de-adenylation, translational repression, 5′-to-3′ mRNA degradation, and de-capping [5, 84]. MicroRNAs are dynamic in their regulatory activities. A single miRNA has the ability to bind to and regulate the expression of sometimes even hundreds of mRNAs. Similarly, multiple miRNA types can bind to a single mRNA target in the 3′UTR and regulate its expression in concert or individually. Computational studies have predicted that more than 50% of human protein–coding genes are regulated by miRNAs [5, 23, 129].

Several studies have described the important role of miRNAs, and deregulation of miRNAs has been implicated to underlie many different pathological conditions [98]. MiR-26a, miR-197, and miR-210 have been characterized as high-risk factors for patients with colorectal cancer (CRC), whereas miR-375 has served as a protective factor [125]. Vascular endothelial cells (VECs) highly express miR125a-5p and miR-125b-5p. Deregulation in the expression of these miRNAs may contribute to vascular dysfunction [64]. MiR-146a is thought to negatively regulate the innate immune response [90]. Diabetes patients with or without diabetic retinopathy (DR) can be differentiated based on the expression levels of miR-93 and miR-152. Both miRNAs showed good diagnostic performance for diabetes and DR [93]. MiR-10 acts as an endogenous inhibitor of the NLRP3 inflammasome. It is able to suppress the activation of NLRP3 (NOD-, LRR, and pyrin domain–containing protein 3) inflammasome and prevent inflammation in a diabetic kidney. In fact, miRNAs play a pivotal role in mediating the regulation of many physiological and pathophysiological processes [22].

## Hypoxia-microRNA crosstalk

Hypoxia signaling influences miRNA expression and activity, and those that respond to hypoxia have been termed hypoxamiRs [28]. However, a bidirectional crosstalk has also been described in which microRNAs can target HIFs and thereby modulate their expression and signaling [10, 97]. Several miRNAs such as miR-6789-5p, miR-98-3p, miR-139-5p, miR-520d-3p and miR-4745-5p [75], miR-210-3p [41, 70, 75], miR-687 [8], miR-155 [10], miR363 [122], miR-19a [1], and miR-146a [101] have been identified as HIF-dependent miRNAs. Except for miR-6789-5p, which was a HIF1-specific miRNA, all of these miRNAs were also identified to be regulated by HIF2. Other HIF2-specific miRNAs have also been described: miR-7-5p, miR-503-5p, miR-503-3p, miR-424-3p, miR-495-3p, and miR-543 [75].

Many of these hypoxia-related miRNAs play critical roles in the regulation of various biological processes. MiR-210 has been widely described in the field of hypoxia signaling and is predominantly upregulated under hypoxic conditions [117]. It regulates mitochondrial function, proliferation, DNA repair, apoptosis, cell migration, and cell cycle [42]. Hypoxia-related miRNAs are of great prognostic value for identifying high-risk patients with various types of cancer, including colorectal cancer (CRC) [125]. Moreover, patients with pancreatic ductal adenocarcinoma (PDAC) have increased expression levels of total miR-30b-5p and peripheral blood plasma exosomal miR-30b-5p compared to healthy individuals. MiR-30b-5p is enriched in hypoxic pancreatic cancer cells and it directly targets and inhibits GJA1 expression in endothelial cells thereby facilitating angiogenesis [13].

Hypoxia can alter the expression levels of microRNAs depending on the cellular and physiological context. MiR-140-3p expression is induced under hypoxia, and its overexpression has been shown to enhance osteo/odontogenic differentiation of hypoxia-treated dental pulp stem cells (DPSCs) by negatively regulating lysine methyltransferase 5B (KMT5B) [131]. MiR-34a expression is induced under hypoxia. MiR-34a targets and represses the expression of Klotho in ARPE-19 cells [121]. PHDs, which target HIFs, may also be targeted by miRNAs. MiR-122, an HIF1-regulated miRNA, was identified to target and repress PHD1 upon hypoxia induction and facilitate liver protection by attenuating liver injury during liver transplantations [48]. Similarly, hypoxia-elevated miR-182-5p has been described to regulate contractile vascular smooth muscle cells (VSMCs). MiR-182-5p acts as a negative regulator of RhoA signaling in VSMCs by repressing RGS5 expression under hypoxia [113].

Hypoxia can also lead to miRNA downregulation. MiR-873-5p is significantly downregulated under hypoxic conditions [24]. HIF1α has been shown to directly bind the tumor suppressor miR-326 promoter and suppress its expression under hypoxic conditions. MiR-326 targets and suppresses ITGA5 levels thereby ameliorating resistance to chemotherapy in triple-negative breast cancer (TNBC) [4]. There is also a bidirectional crosstalk between HIFs and microRNAs. MiR-196b-5p has been reported to target high-mobility group AT-hook 2 (HMGA2) mRNA, thereby inhibiting angiogenic function. Simultaneously, miR-196b-5p is downregulated in endothelial cells as a result of HIF1α regulation. However, by targeting HMGA2, miR-196b-5p may also reduce the expression levels of HIF1α [86]. The tumor suppressor protein P53 inhibits tumor angiogenesis by inducing microRNA-107 expression in human colon cancer cells. MiR-107 targets HIF1β in these cells, reducing its expression and hypoxia signaling [123].Treatment of metastatic clear cell renal cell carcinoma (m-ccRCC)-bearing patients with EGFR tyrosine kinase inhibitors (VEGFR TKIs) as first-line therapy has identified three miRNAs (miR-3529-3p, miR-185-5p, and miR-223-3p) that target HIF-2α and are associated with reduced expression and tumor reduction [51]. These studies represent a growing catalog of possible interactions between HIFs and microRNAs. Such a catalog can be used as part of the effort to establish specific HIF inhibitor regimens. Further investigation of the biological and therapeutic relevance of these associations is warranted.

## MicroRNAs regulate steroidogenesis

Steroid hormones play a critical role in many physiological events [38]. Steroids are essential for immune cell function, cell metabolism, reproduction, salt and water balance, pregnancy, and the body’s response to stress signals. The biochemistry of steroid synthesis has been described in detail [5, 12]. Steroidogenesis involves the concerted activity of steroidogenic enzymes in catalyzing the conversion of cholesterol and the production of different types of steroids depending on the cell type, tissue type, or species. The steroidogenic acute regulatory protein (StAR) facilitates the mobilization of free cholesterol from the cytoplasm into the mitochondria. The enzyme “cytochrome P450scc” (also known as CYP11A1) cleaves the cholesterol side chain to produce pregnenolone, which is then converted to other steroids via oxidation by a series of oxidative enzymes present in both the mitochondria and the endoplasmic reticulum [12].

Five classes of steroid hormones include (1) glucocorticoids (with cortisol being the main glucocorticoid in humans and corticosterone in rodents, reptiles, and birds), (2) mineralocorticoids (with aldosterone being the main mineralocorticoids in humans) (3) estrogens such as estrone and estradiol, (4) progestins such as progesterone, and (5) androgens such as dihydrotestosterone and testosterone [5]. In addition to the canonical tissue-specific trophic hormone regulation of steroid hormone biosynthesis and the transcriptional and translational events underlying the regulation of chronic steroidogenesis [38], there are several other mechanisms of regulation of the steroidogenic pathway that are currently under investigation. These include post-transcriptional and post-translational regulatory mechanisms such as phosphorylation/dephosphorylation, as well as miRNA and protein–protein interactions [5].

MiRNAs play a critical role in the regulation of steroidogenesis. Using computational studies, several miRNAs have been identified that bind to specific sequences in the 3′UTR of various target mRNAs of steroidogenic enzymes and thus modulate their expression. These miRNAs are currently being validated in various experimental conditions and models [10, 119]. MiRNAs regulate different steps of corticosteroid biosynthesis. Downregulation of miRNA levels by Dicer1 knockdown induced the expression of several corticosteroidogenic genes, including CYP11A1, CYP11B1, CYP11B2, CYP17A1, and CYP21A. Increased expression of these genes was associated with increased levels of steroids such as corticosterone, aldosterone, and 18-hydroxycorticosterone. In this study, miR-125a-5p and miR-125b-5p were shown to directly suppress CYP11B2 expression levels in vitro [88]. MiR-132 has been shown to modulate steroidogenesis. It represses StAR protein expression while inducing 20α-HSD enzyme mRNA levels, leading to the production of biologically inactive steroids [40]. Together with collaborators, we have previously described the dysregulation of adrenal miRNAs in different forms of Cushing’s syndrome (CS). Analysis of human adrenal tissue revealed that miR-1247-5p and miR-379-5p are significantly associated with different CS subtypes [106]. Moreover, even circulating miRNAs are possible hypercortisolism biomarkers in different CS subtypes [105, 106]. MiR-150 negatively modulates steroidogenesis of Leydig cells in mice by targeting and repressing the expression of StAR [27]. Taken together, repression of steroidogenic enzyme expression and consequently steroid hormone synthesis by the action of miRNAs may prove critical in disrupting the reproductive system and normal physiological processes, as well as other developmental processes in which optimal steroid levels are essential. The effect of miRNAs on steroidogenesis may be crucial for ameliorating disorders associated with impaired steroid production. Understanding the role of miRNAs in the regulation of steroidogenesis is therefore of paramount importance.

## Hypoxia regulates steroidogenesis via a crosstalk with microRNA

A number of studies with zebra fish, mouse, and different cell lines revealed the activity of hypoxia/HIF on steroidogenesis with a resultant disruption of aldosterone and glucocorticoid biosynthesis [53, 68, 114, 124]. Our group has recently described the effect of hypoxia pathway proteins on steroidogenesis. We reported that cell-specific overexpression of HIF1α in mouse adrenal cortical cells resulted in the impaired expression of essential steroidogenic enzymes and, consequently, steroids [118].

Steroidogenesis thrives on cholesterol availability, and although several reports have suggested a disruptive action on normal cholesterol biosynthesis and lipid accumulation as a result of HIF pathway activity, findings from our mouse model revealed a reduction in the size of lipid droplets in the adrenal glands associated with increased steroid production [118]. Such a perceived contradiction in the differential activity of the HIF pathway in regulating cholesterol metabolism and steroidogenesis may point to the presence of other HIF-independent regulators under conditions of complete hypoxia, which are absent in our mouse model. Sterol regulatory element binding protein 2 (SREBP2) has recently been described as a central transcription factor underlying mammalian sterol production. SREBP2 is degraded under hypoxia in a HIF-independent manner, ultimately distorting normal sterol production [21].

**Fig. 2:**
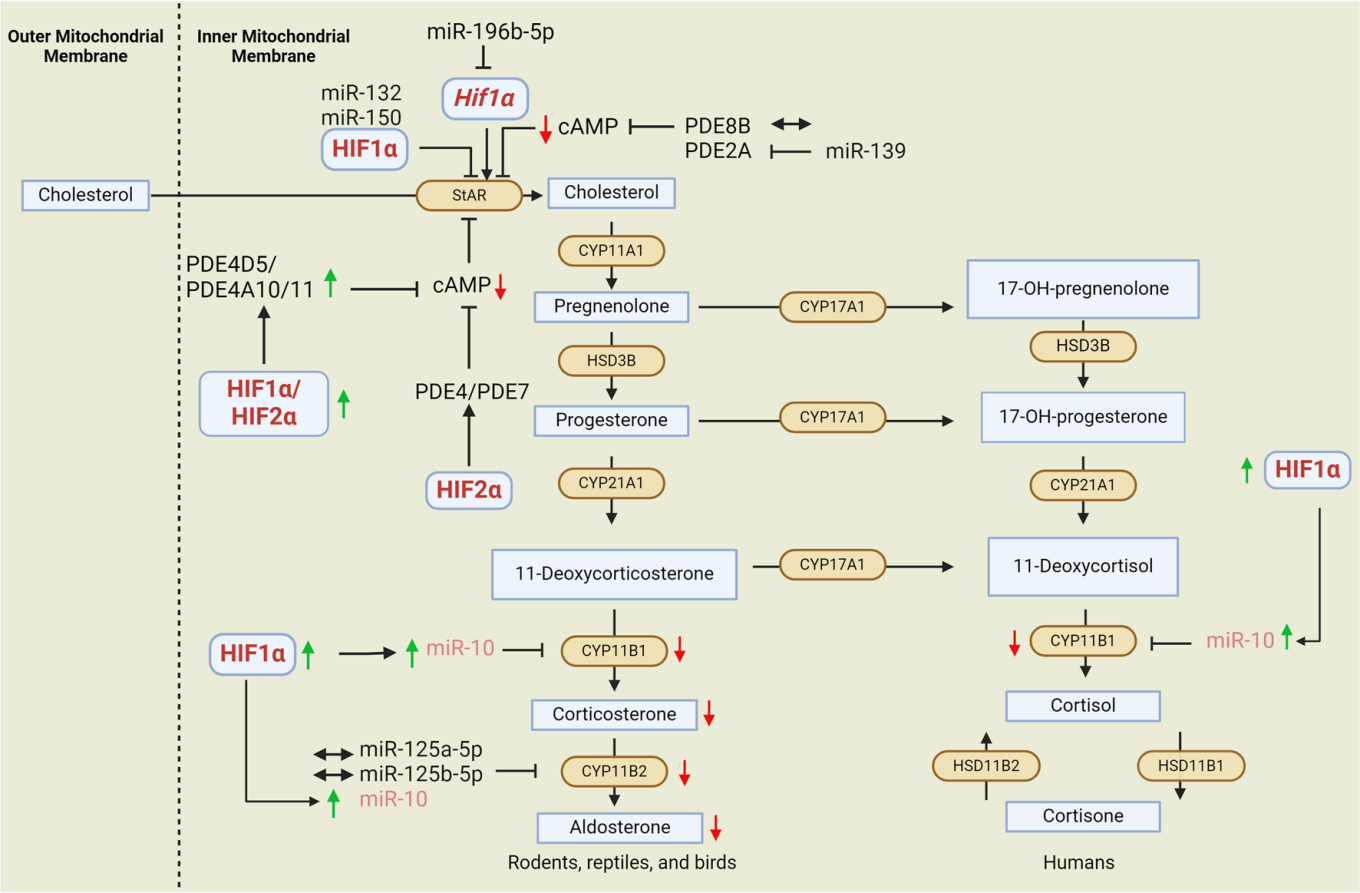
Hypoxia pathway proteins-microRNA-phosphodiesterase crosstalk in steroidogenesis regulation. Hypoxia regulates steroid production through either direct binding of HIFs to the HREs in the promoter of steroidogenic enzymes or by a crosstalk of HIFs with other regulators such as microRNAs (miRNAs) and phosphodiesterases (PDE) (see further). The expression and activity of steroidogenic enzymes as well as steroidogenesis are regulated by miRNAs and PDE. MiRNAs and PDE are also regulated by each other, which ultimately impacts steroidogenesis either positively or negatively. MiRNAs may also target *Hif1α*. Hypoxia is able to alter the expression and activity of miRNAs and PDE, which ultimately affects steroid production

Mechanistically, the effect of HIF on steroidogenesis is possible through different mechanisms. HIFs can directly bind to the promoters of steroidogenic enzyme genes and regulate their transcription. For example, HIF1α has been reported to directly repress the expression of the mitochondrial cholesterol transporter StAR by directly binding to 3 different HREs in the StAR promoter [114] (see Fig. 2). Admittedly, this study represents one of the very few and unusual examples of direct HIF-mediated suppression of gene expression. Other possible mechanisms such as the displacement of other important transcriptional activators, HIF interaction with specific epigenetic factors, and the recruitment of co-repressors to HIF may therefore be involved in mediating the HIF1 negative regulation of StAR expression and other gene expressions in general [74, 95].

MiRNAs are potent epigenetic regulators of gene expression [26, 81, 129]. As described in other established reports where HIF negatively regulates different biological processes, HIF may also mechanistically regulate steroidogenesis through its established crosstalk with miRNAs [37, 48, 113, 131]. Using in silico algorithms such as PicTar (http://pictar.bio.nyu.edu/), TargetScan (http://www.targetscan.org/), and Sanger microRNA target (http://microrna.sanger.ac.uk/), potential miRNAs of steroidogenic enzymes can be identified. In this way, the upregulation of miRNAs targeting mRNAs of steroidogenic enzymes can potentially elucidate the paradox of the negative regulation of steroidogenesis upon hypoxia/HIF1α upregulation [57, 80].

Recent studies have investigated the effect of hypoxia on the activity of miRNAs in the regulation of steroidogenesis. Hypoxia-inducible miR-10 was reported to be a negative regulator of two important steroidogenic enzymes, namely CYP11B1 and CYP11B2, repressing their expression in H295R, a human adrenocortical carcinoma cell line [80]. CYP19A1 (*Aromatase*) was reported to be downregulated as a result of the induction of a HIF1-inducible miRNA, miR-98 [127].

Hitherto, most hypoxia-microRNA–related studies on steroidogenesis have been investigated across in vitro experimental models [80]. It would be of great interest to conduct studies in models that closely reflect real physiological conditions, such as in steroidogenic tissues and in vivo experimental models, to corroborate the first few existing findings on the impact of hypoxia-microRNA crosstalk and steroidogenesis regulation. To further elucidate the exact molecular mechanisms by which hypoxia regulates steroidogenesis, under hypoxia, the experimental validation of other unexplored miRNAs that have already been predicted to target steroidogenic enzymes and regulate steroidogenesis is worth pursuing in future studies.

## Hypoxia-inducible factor prolyl hydroxylase inhibitors (PHDi) and steroidogenesis

Given the critical role of hypoxia/HIFs in steroid production as described in the previous sections, it is important and appropriate to highlight some of the currently available opportunities to stabilize HIFs and thereby modulate steroid production. Several HIF stabilizers with potential clinical relevance have been approved in the past and/or are currently under clinical trials [62, 116, 130]. Although most of these drugs are primarily designed to treat conditions such as anemia of various etiologies, ischemia, and inflammation, not much is known about their effects on steroid production. Most of the drugs currently available to inhibit the activity of PHD include the HIF competitors, iron chelators, iron displacers, and α-ketoglutarate mimetics. Drugs such as roxadustat, dimethyloxalylglycine (DMOG), enarodustat, daprodustat, desidustat or ZYAN1 (Zydus), and vadadustat belong to the group of α-ketoglutarate mimetics. Deferoxamine (DFO) and ciclopirox are metal chelators. Metal ions such as cobalt, manganese, nickel, and zinc are iron displacers [82]. The most clinically advanced of these drugs are the 2-OG derivatives, which compete with 2-oxoglutarate (2-OG) for binding sites in PHDs, rendering them inactive [116].

It is worth noting that some of the hypoxia-inducible factor prolyl hydroxylase inhibitors (HIF-PHIs) may affect cholesterol levels, probably directly related to the fact that HIF has been shown to reduce cholesterol synthesis [43]. Roxadustat treatment has been shown to result in lower LDL cholesterol levels [14, 62, 83]. Conversely, no clear correlations were observed for the effect of the oral hypoxia–inducible factor prolyl hydroxylase inhibitor vadadustat on cholesterol levels after use in phase 2 clinical trials [77]. The explanations for the observed ambivalence in the role of different HIF-PHIs on cholesterol levels remain poorly understood and require further investigation [61]. The extent to which non-erythropoietic pathways are targeted in patients receiving these treatments remains a puzzle, and the use of these interventions is not without potential risks [54]. The lack of mechanistic specificity in the action of these drugs may interfere with normal steroid production even after HIFs have been stabilized. Future studies delineating the full effects of these drugs on steroidogenesis and cholesterol metabolism are warranted.

The effect of clinically approved roxadustat on steroidogenesis in immortalized murine KK1 granulosa cells has been reported. Treatment of these cells with roxadustat resulted in a biphasic StAR protein expression response. Specifically, at lower roxadustat concentrations, the expression of the StAR protein was upregulated; however, with increased doses of roxadustat and consequent enhancement in HIF1α stabilization, StAR expression levels were significantly reduced indicating an inverse relationship between HIF1α and StAR expression levels [29]. The role of cobalt chloride (CoCl2)-induced hypoxia on steroid production in Leydig cell–derived MA-10 cells has been reported, with CoCl2 treatment leading to the inhibition of progesterone production as well as direct inhibition of P450scc mRNA levels, which could be attributed to the resultant increased HIF1α activity [55]. Karimi and colleagues also examined how melatonin treatment affects steroidogenesis following hypoxia induction in TM3 Leydig cells. Treatment of these cells with CoCl2 resulted in decreased Hsd3b1 and StAR mRNA/protein expression [50]. In contrast, administration of dimethyloxalylglycine (DMOG) led to an increase in plasma corticosterone as early as 5 min after airpuff startle (APS), an acute stressor, which was abolished by 2 h [31].

However, the use of these drugs is not without limitations due to unspecific PHD binding and possible off-target effects of α-ketoglutarate mimetics, as there are more than 50 α-ketoglutarate-dependent oxygenases in the human genome. Also, the toxicity of cobalt and the involvement of iron in many cellular processes and its displacement or chelation can have dire consequences. A great concern has also been directed to their use, as the prolonged stabilization of HIFs may result in modulations in the expression of arrays of HIF target genes and may be implicated in the etiology of pathologies such as increased risk of hyperkalemia and iron deficiency development. Consequently, further research aimed at modifying the activities and specificities of these drugs, as well as reducing their side effects, is of paramount importance in order to maximize their therapeutic efficacy and maybe even steroidogenesis regulatory potential [116].

The regulation of PHDs by microRNAs has been suggested and also requires further investigation to better understand the possibilities around the miR-PHD axis as a mechanism for steroidogenesis regulation. In this regard, Chen and colleagues have already shown that miR-17 is able to target prolyl hydroxylases 2 (PHD2) in its 3′-UTR and that overexpression of miR-17 in pulmonary artery smooth muscle cells (PASMC) leads to the decreased expression of PHD2 and induction of HIF1α protein levels [15]. Further research on the repressive potential of microRNAs on PHD expression, which would ultimately promote HIF1 activity, as well as the implications for steroidogenesis is warranted.

## Phosphodiesterases regulate adrenal cAMP-dependent steroidogenesis

Steroidogenesis is also induced by the cAMP signaling pathway downstream of the ACTH receptor in the adrenal gland. Stimulation of the G-protein-coupled ACTH receptor leads to activation of adenylate cyclases (ACs), which generate the intracellular second messenger cAMP. Further activation of cAMP-dependent protein kinase A (PKA) promotes steroidogenesis by both acute and chronic mechanisms [103]. The acute mechanism involves the phosphorylation of StAR, which facilitates the transport of cholesterol into the mitochondria for steroid hormone synthesis [67]. The chronic mechanism involves the transcriptional regulation of steroidogenic enzymes, which is mediated by the phosphorylation of transcription factors by cAMP-dependent PKA [46].

The spatial and temporal concentration of cellular cAMP is controlled by the interplay between its production by ACs and its hydrolysis by phosphodiesterases (PDE). In mammalian species, 11 families of PDE have been described, each characterized by distinct kinetics, regulatory partners, and tissue distribution [109]. PDE play a pivotal role in governing the intracellular levels of both cAMP and cGMP, as well as the respective signaling pathways and downstream targets. The adrenal cortex expresses several isoforms of PDEs [58]. Here, we will focus on PDEs which are associated with adrenal gland–related diseases. Current research projects in our organization are focused on investigating a potential link between PDE-HIF and steroidogenesis (see further).

### PDE2

The predominant PDE isoform in adrenal tissue is PDE2A, which is a dual-substrate phosphodiesterase capable of degrading both cAMP and cGMP with similar affinities (35 µM for cAMP and 11 µM for cGMP). PDE2A can be stimulated via cGMP-binding to the regulatory GAF-B domain thereby enhancing the cAMP hydrolyses up to 30-fold [69]. Thus, PDE2 induces a so-called negative cGMP/cAMP crosstalk, which has been described in several cell types, including cardiomyocytes and adrenal zona glomerulosa cells [91, 110]. In the heart, the cGMP-dependent PDE2 activation as well as PDE2 overexpression resulted in reduced catecholamine-induced arrhythmia after myocardial infarction [11, 71, 108]. In situ hybridization showed a strong expression of PDE2 in the zona glomerulosa cells of the adrenal gland [102]. PDE2 was the first cAMP degrading enzyme that was described to directly regulate steroid synthesis. In zona glomerulosa cells, the A-type natriuretic peptide (ANP) induced cGMP generation upon NPRA receptor stimulation. The following cGMP-dependent activation of PDE2 resulted in dose-dependent cAMP hydrolysis and attenuation of aldosterone secretion [65]. The ANP effect in human glomerulosa cells was prevented by PDE2 inhibition with EHNA (erythron-9-[2-hydroxo-3-nonyl] adenine) [17]. The influence of the simultaneous activation of the cGMP-dependent protein kinase G (PKG) on aldosterone secretion is controversially discussed. In rat glomerulosa cells, the direct PKG stimulation with the cGMP analog 8-pCPT-cGMP promoted aldosterone synthesis [100], whereas the injection of ANP reduced serum aldosterone levels in WT mice but not in mice with genetic PKGII deletion [25]. The consequences of cGMP-dependent PDE2 stimulation are cell specific, but mediate beneficial effects in cardiomyocytes and adrenal glomerulosa cells [11, 107]

### PDE8

The PDE8 family also plays a role in adrenal steroidogenesis. The two isoforms PDE8A and PDE8B, encoded by different genes, specifically degrade cAMP with high affinity (*K*_m_ = 0.15 µM) without affecting cGMP. While PDE8A is expressed only at low levels in a subpopulation of adrenal zona fasciculata cells, transcripts of PDE8B are highly abundant throughout the fasciculata layers [34, 104]. Using isoform-specific knockout mice, PDE8B was shown to control the corticosterone production in the adrenal gland by regulating the activity of cAMP-dependent PKA and the expression of StAR protein [103, 104]. PDE8B knockout mice showed elevated urinary corticosterone levels due to adrenal hypersensitivity to ACTH with enhanced steroid production. Specific PDE8 inhibition increases acute adrenal steroid production in adrenal Y-1 cells in vitro as well as basal adrenal steroidogenesis in vivo [104]. A point mutation of PDE8B was identified in patients with adrenal hyperplasia [35]. Heterogeneous expression of the mutant in HEK cells increased cellular cAMP levels, suggesting that impaired PDE8B function, amongst other factors, may contribute to the development of adrenal hyperplasia.

### PDE11

PDE11 was the last identified enzyme of the PDE family and is encoded by one single gene PDE11A with four transcriptional splicing variants (PDE11A1 to PDE11A4) [66]. Like PDE2, PDE11A is a dual-specificity phosphodiesterase that catalyzes the hydrolysis of both cAMP and cGMP with similar affinities. The function of its regulatory GAF-domains remains unclear. PDE11A is expressed in several endocrine tissues, including the adrenal gland [18]. Mice with global genetic PDE11A deletion showed only a reduced enzyme expression but displayed adrenal subcapsular hyperplasia with preserved fetal-like proteins in the inner adrenal cortex [60]. In contrast to WT mice, PDE11A KO mice were not responsive to a low dose dexamethasone treatment followed by the suppression of corticosterone secretion. In tissue lysates from adrenal glands, PDE11B deletion resulted in enhanced cAMP concentrations and increased PKA activity [60]. Human PDE11A defects are associated to adrenal diseases, after a genome-wide study identified the chromosome 2q31.2 PDE11A gene locus in 2006 [33]. Three PDE11A mutations have been reported in Cushing syndrome patients with primary pigmented nodular or isolated micronodular adrenocortical disease. Furthermore, single-nucleotide polymorphisms, truncation mutations, and missense substitutions in the PDE11A sequence have been linked to adrenocortical tumors [52].

### PDE3

PDE3 is a family of phosphodiesterases that hydrolyze both second messengers, but with significantly higher catalytic rates for cAMP than for cGMP. At the same time, PDE3 has similar high affinities for cAMP and cGMP, resulting in competitive inhibition of cAMP hydrolysis in the presence of cGMP. Therefore, PDE3 is often referred to as the cGMP-inhibited PDE [76]. Within the PDE3 family, two genes—PDE3A and PDE3B—have been identified, including three transcriptional isoforms of PDE3A that differ in the length of the N-terminal sequence. PDE3 is mainly expressed in the myocardium with minor transcripts in other tissues, such as the adrenal gland [58], but few studies have reported the influence of PDE3 on steroidogenesis. In human adrenocortical H295R cells, specific PDE3 inhibition with cilostamide decreased angiotensin II–stimulated aldosterone levels, suggesting the idea of targeting PDE3 in the treatment of hypertension [16]. Another study demonstrated the inhibition of ACTH-induced cortisol secretion by leptin in human adrenocortical carcinoma cells (NCI-H295). The leptin effect was downstream mediated by PDE3-induced cAMP degradation, since selective PDE3 inhibition abrogated the reduction of cortisol synthesis [36]. Two rare germline variants in the PDE3B gene were identified in patients with primary aldosteronism and bilateral adrenal hyperplasia. The PDE3B mutants mediated a significant reduction in PDE3B expression levels in transfected HEK cells. Another germline nonsense variant of PDE3B was discovered in a child with an adrenocortical tumor [85].

## Phosphodiesterases are regulated by miRNA

There is evidence that microRNAs play a role in the regulation of phosphodiesterases, which are also expressed in the adrenal gland. Previous studies have shown that PDE2 is the host gene for miR-139, which is in the second intron of the PDE2A gene sequence. Accordingly, a strong correlation was observed between miR-139 and PDE2A expression levels, as well as DNA methylation in the PDE2A promoter [94, 115]. In human glioblastoma tissues, miR-139 expression was associated with PDE2A transcription and shared correlation of expression levels. However, the overexpression of miR-139 increased the mRNA levels of PDE2A and pre-miR-139 in glioblastoma cell lines. In addition to the dependency of expression, both miR-139 and PDE2A act synergistically in glioblastoma cells by repressing detrimental Wnt/β-catenin signaling and reducing the tumorigenesis of gliomas [63].

In silico prediction identified 67 PDE3A-related miRNAs, including miR-27a-3p and miR-222-3p. Expression of these miRNAs in a human endothelial cell line resulted in a significant reduction of PDE3A expression [126]. In suppressive regulatory T cells (*T*_reg_), it was shown that miR-142-5p directly targets the PDE3B mRNA, limiting PDE3B protein levels. Thereby, miR-142-5p regulated intracellular cAMP levels and controlled *T*_reg_ suppressive functions [3]. Interestingly, the *T*_reg_-specific deletion of miR-142 in mice led to enhanced PDE3 expression in *T*_reg_ cells, impaired suppressive *T*_reg_ function and lethal multisystem autoimmunity while PDE3 inhibition with cilostamide restored *T*_reg_ function and prevents autoimmune disease [3].

The influence of miRNAs on PDE8A was investigated in immune and cancer cells. Thus, miR-145-5p was shown to regulate PDE8 expression levels by targeting the 3′UTR of the PDE8A mRNA transcript. Accordingly, PDE8A expression negatively correlates with miR-145-5p levels during the differentiation of monocytes into macrophages [9]. Another study showed the association of PDE8A with miR-33a in glioma cells. In vivo inhibition of miR-33a using a locked nucleic acid (LNA) enhanced the PDE8A expression and reduced tumor growth in mice [112].

Using computational-driven methods, intronic regions of the oncogene musashi RNA binding protein 1 (MSI1) were analyzed for potential precursor miRNAs. The study identified the new precursor miRNA MSM-3, which is expressed in several cancerous cell lines as well as human breast cancer tissue. PDE11 was confirmed to be a direct target of MSM3-3p addressing the 3′UTR region of PDE11A. Cancer cell lines with high miR MSM3-3p expression displayed reduced PDE11 levels [56]. In microarray analysis of miRNA and related genes in colorectal cancer stromal tissue, PDE11A was found to be highly inversely correlated with two other miRNAs, namely miR-18a and miR-19a [79].

## Hypoxia-phosphodiesterase crosstalk, a possible mechanism in the regulation of steroidogenesis?

The role of some PDE isoforms in steroidogenesis has been described in earlier sections of this review, and several isoforms are expressed in the adrenal gland, including the ubiquitous phosphodiesterase 5 [45]. Other PDE isoforms have been identified in normal adrenal glands, cortisol-producing adenomas, and macro-nodular adrenocortical hyperplasia, such as PDE11A, PDE2A, PDE8E, and PDE8B [30, 34].

Hypoxia pathway proteins modulate phosphodiesterase expression and activity, while modulation of the PDE activity by hypoxia affects downstream signaling pathways. At the molecular level, HIF2α has been shown to induce the expression of cAMP-specific phosphodiesterases, which was confirmed by the inhibition of HIF prolyl hydroxylases [87]. It was shown that the induction of cAMP-specific phosphodiesterase expression and activity (including PDE4 and 7) in a HIF2α-dependent manner ultimately leads to glucagon signaling and sensitivity blockade in mouse and human hepatocytes. These findings suggest a PHD-dependent regulation of PDE via HIF2α [87] (see Fig. 2).

Hypoxia has been described to activate the cAMP/PKA pathway by inducing the expression of the adenylyl cyclase system of enzymes that facilitate the production of cAMP. The cAMP/PKA pathway is important for cells to adapt to hypoxia. Studies have shown that HIF regulation of adenylyl cyclases 6 and 7 led to the induction of cancer-associated cAMP and PKA signaling despite increased activity of PDEs that control cAMP degradation [99]. In human pulmonary artery smooth muscle cells (hPASM cells), hypoxia was shown to mediate a brief induction of PDE4B2 expression, which later peaked after day 7, with concomitant upregulation of both intracellular cAMP levels without altering total cAMP PDE3 or cAMP PDE4 activity. However, in this study, hypoxia also upregulated PDE4D5 and PDE4A10/11 levels over a 14-day period [73]. The effects of PDE-selective inhibitors in the treatment of various physiological and pathological conditions, including steroidogenesis, have been described. Vardenafil, a phosphodiesterase 5 inhibitor, has been shown to promote angiogenesis via a protein kinase G-dependent HIF1/VEGF pathway [92]. Tadalafil, a type 5 phosphodiesterase inhibitor, increases salivary cortisol levels in response to stress [20].

Hypoxia appears to upregulate both cAMP levels and PDE expression depending on the cellular context or tissue type. In addition, severe hypoxia has been reported to negatively affect protein kinase A phosphorylation [47]. The cAMP/PKA pathway, which can be regulated by PDE, is critical for StAR expression and activity [53]. Given that both hypoxia and PDE affect steroidogenesis as described in the previous discussion, and that hypoxia can affect PDE activity, we propose that there is a crosstalk between PDE and hypoxia in the regulation of steroidogenesis. Hypoxia induction of PDE may result in the modulation of cAMP levels required to regulate certain aspects of steroidogenesis. Studies elucidating such a relationship are scarce and warranted. Elucidation of such crosstalk may provide great insight into further delineation of other molecular mechanisms by which hypoxia may regulate steroidogenesis in steroid-producing cells. Findings from such studies may potentially point to novel therapeutic avenues.

## Modulation of miRNA expression by second messengers and steroid hormones

Interestingly, some studies have also reported the modulation of miRNA expression in response to hormonal treatment and second messenger activity. Several miRNAs expressed in the adrenal gland and other steroidogenic cells are regulated by various hormones and the second messenger cAMP. This regulation ultimately affects the steroid output of these cells/tissues. The miRNA-132 expression level is increased in undisturbed adrenal glands following adrenocorticotropic hormone (ACTH) treatment and in Y1 adrenocortical cells following cAMP treatment [40]. MiRNA-455, miRNA-125a, and miRNA-125b expression levels are reduced in rat adrenals treated with ACTH in vivo as well as in MLTC-1 cells and primary rat granulosa cells treated with cyclic AMP (cAMP) [39]. Treatment of rats with ACTH, dexamethasone, or 17α-E2 affects the expression of several miRNAs. Specifically in the adrenal glands, miRNA-182, miRNA-212, miRNA-132, miRNA-183, and miRNA-96 expression levels are upregulated while miRNA-214, miRNA-27a, miRNA-503, and miRNA-466b expression levels are downregulated upon ACTH treatment. In response to treatment with 17α-E2, the expression levels of miRNA-200b, miRNA27a, miR-125b, miR-122, miRNA-214, miR-138, miRNA-466b, and miRNA-503 are downregulated, whereas the expression levels of miRNA-370, miR-212, miRNA-377, miRNA-183, miRNA-96, miRNA-370, and miRNA-132 are upregulated. Treatment with dexamethasone only led to an increased miRNA-183 expression level and a decrease in the expression levels of miR-19a, miRNA-200b, miRNA27a, miR-122, and miRNA-466b [38].

There is an interaction between pituitary-adrenal axis hormones and circulating microRNAs associated with the adrenal cortex. Hormonal treatment of 10 individuals with either dexamethasone or tetracosactide (adrenocorticotropin) altered the expression profile of miRNAs in human plasma samples. Hsa-miR-27a expression was suppressed by adrenocorticotropin, whereas dexamethasone induced its expression. This effect was also recapitulated in NCI-H295R cells treated with dexamethasone. Since adrenal mass dexamethasone suppression and ACTH stimulation assays are very important functional endocrine diagnostic tests, knowledge of microRNA expression patterns as a result of these assays may be particularly useful in screening for adrenal malignancies that would ultimately affect normal adrenocortical hormone production. [44]. Sex steroid hormones also regulate microRNA expression. Treatment of EEC cell lines with estrogen-induced miR-196a-5p expression and increased the viability of these cells by targeting Forkhead box protein O1 (FOXO1) [132]. Progesterone induces miR-145/143 expression leading to the inhibition of cyclin D2 expression and proliferation of endometrial epithelial cells [128].

These studies reveal a complex interplay between hormones, second messengers, microRNAs and steroidogenesis. MicroRNAs under the control of various hormones are important in mediating the regulatory control of these hormones on steroidogenesis. Increased steroid levels may be attenuated by a possible feedback mechanism that induces the expression of microRNAs to negatively control the expression of steroid biosynthetic enzymes. Future studies are needed to decipher the precise molecular mechanisms underlying the interaction between microRNAs and these different hormones, the role of specific hormone-regulated miRNAs in adrenal cells, the consequences for the regulation of steroidogenesis, and their biological relevance.

## Conclusions and perspectives

Pathophysiological hypoxia can occur in a variety of environmental and pathological conditions and may well adversely affect systemic processes including the nervous, cardiovascular, and endocrine systems [78]; the latter encompassing the adrenal, pancreatic, thyroid, and ovarian glands, which play central roles in maintaining the body’s homeostasis [59]. Hypoxia/HIF has been widely considered systemically undruggable due to its critical role in regulating myriad physiological processes. Therefore, the focus is now shifting to targeting downstream effectors of HIF as a means to generate novel and less complicated therapies [59]. Since miRNAs and PDE may be such downstream effectors, and there is bidirectional crosstalk between both, pharmacological targeting of hypoxia-inducible miRNAs may provide potential interventions for ameliorating several diseases [48]. Conversely, since the hypoxia pathway proteins play a vital role in the adrenal gland, uncontrolled deregulation of their expression or related machinery may negatively affect normal adrenal function and lead to various adrenal-related disorders. In addition, the therapeutic use of HIF prolyl hydroxylase inhibitors (PHDi) must be carefully controlled and their effects on steroidogenesis well considered. Further research is therefore warranted to better understand these diverse effects, including potential links between HIF-PDE and steroidogenesis, and to translate them into new potential therapies.

## Data availability

No datasets were generated or analyzed during the current study.
